# Evaluation of the hypothalamic-pituitary-adrenal axis and its relationship with central respiratory dysfunction in children with Prader-Willi syndrome

**DOI:** 10.1186/s13023-015-0312-z

**Published:** 2015-09-02

**Authors:** Veronique Beauloye, K. Dhondt, W. Buysse, A. Nyakasane, F. Zech, J. De Schepper, S. Van Aken, K. De Waele, M. Craen, I. Gies, I. Francois, D. Beckers, A. Desloovere, G. Francois, M. Cools

**Affiliations:** Unité d’Endocrinologie pédiatrique, Cliniques Universitaires Saint-Luc, Université catholique de Louvain, avenue Hippocrate 10/1300, Brussels, B-1200 Belgium; Department of Pediatrics, Division of Child Neurology and Metabolism, Pediatric sleep center, Ghent University Hospital, Ghent, Belgium; Department of Pediatrics, Division of Pediatric Endocrinology, Ghent University Hospital and Ghent University, Ghent, Belgium; IREC, Université Catholique de Louvain, Brussels, Belgium; Department of Pediatrics, Division of Ped Endocrinology, UZ Brussel, Brussels, Belgium; Department of Pediatrics, Division of Ped Endocrinology, KULeuven, Leuven, Belgium; Department of Pediatrics, Division of Ped Endocrinology, CHU Mont-Godinne-Dinant, Université Catholique de Louvain, Yvoir, Belgium; Unité de sommeil, Cliniques Universitaires Saint-Luc, Université catholique de Louvain, Brussels, Belgium

**Keywords:** Prader-Willi, Adrenal insufficiency, Sleep disorders

## Abstract

**Background:**

Children with Prader-Willi Syndrome (PWS) have been considered at risk for central adrenal insufficiency (CAI). Hypothalamic dysregulation has been proposed as a common mechanism underlying both stress-induced CAI and central respiratory dysfunction during sleep.

**Objective:**

To evaluate CAI and sleep-related breathing disorders in PWS children.

**Patients and methods:**

Retrospective study of cortisol response following either insulin tolerance test (ITT) or glucagon test (GT) in 20 PWS children, and comparison with 33 non- Growth Hormone deficient (GHD) controls. Correlation between sleep related breathing disorders and cortisol response in 11 PWS children who received both investigations.

**Results:**

In PWS children, the cortisol peak value showed a significant, inverse correlation with age (Kendall’s τ = -0.411; *p* = 0.012). A similar though non-significant correlation was present between cortisol increase and age (τ = -0.232; *p* = 0.16). Similar correlations were found in controls. In only 1 of 20 PWS children (5 %), ITT was suggestive of CAI. Four patients had an elevated central apnea index but they all exhibited a normal cortisol response. No relationship was found between peak cortisol or cortisol increase and central apnea index (respectively *p* = 0.94 and *p* = 0.14) or the other studied polysomnography (PSG) parameters.

**Conclusions:**

CAI assessed by ITT/GT is rare in PWS children. Our data do not support a link between CAI and central respiratory dysregulation.

## Background

The Prader-Willi Syndrome (PWS) is a rare, complex neurogenic disorder caused by the loss of expression of the paternally expressed genes from the 15q11-q13 region [1]. The clinical symptoms are age-related: fetuses show a marked decline of movements in utero, position abnormalities or polyhydramnios. Shortly after birth and during the first year of life severe hypotonia, lethargy, breathing difficulties and failure to thrive are prominent. During infancy, growth deceleration and developmental delay become evident, together with extreme hyperphagia and a low metabolic rate, causing important obesity. Standard treatment is controlled diet, regular physical exercise and human recombinant growth hormone (GH), which normalizes growth and improves exercise capacity, physical strength, body composition and fat regulation [2, 3].

A dysfunctional hypothalamic-pituitary axis is assumed to underlie several clinical features such as hyperphagia, hypogonadism, aberrant energy regulation, inefficient GH secretion and abnormal temperature regulation. Likewise, hypothalamic dysfunction is thought to be responsible for the disturbed hypoxic ventilatory response commonly seen in these patients. This, in combination with obesity, respiratory muscle weakness, craniofacial abnormalities and adenotonsillar hypertrophy, is thought to lead to the well-known sleep-related breathing disorders (SRBD) in PWS, including central (CA) and obstructive (OA) apneas, and hypopneas. Whereas OA are strongly related to BMI, and are mostly seen in older, obese PWS patients, CA more commonly occur in non-obese, prepubertal PWS children and have not been related to body weight or BMI. In combination with the impaired or, in some cases, even absent response towards hypoxia and hypercapnia, CA are thought to result from a central dysfunctional respiratory control, already present at an early age [4–8].

In addition, young PWS patients have an increased risk of sudden death, especially during sleep. In a series of 64 PWS patients up to nineteen years of age, the main cause of death (61 %) was a respiratory disorder - an upper respiratory tract infection in the majority (44 %) of cases, and suffocation or sudden death during sleep in the remaining group (17 %), independent of GH treatment. The median age at death was 3 years [9]. The cause of sudden death in PWS is not known. de Lind van Wijngaarden et al. [10] suggested that an unexpected and unexplainable death during sleep in children with PWS could be caused by a stress-induced central adrenal insufficiency (CAI), resulting from a dysfunction of the hypothalamic-pituitary-hormonal axis. This could explain the typical lack of symptoms during illness, the higher pain threshold and the low adrenal weight reported during biopsy in patients with PWS. In this study, an inadequate ACTH response to overnight single-dose metyrapone was noticed in 60 % of 25 children with PWS. At baseline, the CA index was higher in those PWS children with CAI compared to those without. After administration of metyrapone, the CA index increased more in children with CAI as compared to children with normal adrenal function, suggesting a link between CAI and sleep-related breathing disorders (SRBD) probably due to a common hypothalamic dysfunction [11]. Other studies, based on low-dose (LDST) and high-dose (HDST) ACTH tests and insulin tolerance tests (ITT), did not confirm these data and described a lower prevalence of CAI in PWS children and adults, ranging between 0 to 14 % [12–15].

In order to gain further insight in the possible relationship between CAI and SRBD in children with PWS, we decided to retrospectively analyze the presence of CAI, based on ITT and GT performed within the context of a global assessment at start of GH therapy in Belgian children with PWS. Subsequent analysis of polysomnographies (PSG) performed in a subset of these children allowed evaluation of SRBD and provided argumentation concerning an eventual underlying central hypothalamic dysfunction, responsible for both CAI and SRBD.

## Methods

Results of ITT and/or GT obtained in 20 PWS (genetically confirmed) children, followed in the period 1997-2012 at different tertiary care centers in Belgium, were retrospectively reviewed. ITT and/or GT were performed before the start of GH treatment in 15 patients. Five PWS patients underwent an ITT and/or GT after the start of GH therapy, but with a one week GH-free wash-out period. ITT and GT had been performed according to previously described protocols [16]. For comparison, results of ITT or GT, obtained in 33 non-GHD otherwise healthy children who were evaluated for short stature in the past three years at the Ghent University Hospital and the Cliniques Universitaires Saint-Luc, were included as controls. BMI was calculated as kg/m2 and expressed as z-score, adjusted for age and sex, using the Cole BMI reference data [17]. GH deficiency (GHD) was diagnosed on the basis of local cut-off values for ITT and GT, and varied according to the used assay at the time of testing.

Serum cortisol levels were determined using local routine laboratory assays and were retrieved retrospectively. A cortisol level exceeding 19.94 μg/dL (550 nM) and/or an increase (calculated between the lowest cortisol and the highest cortisol level during the stimulation test) greater than 9.0 μg/dL (250 nM) were considered as a sufficient cortisol response for both ITT and GT [18]. No direct ACTH measurement was performed; the definition of CAI was based on the interpretation of cortisol responses during ITT or GT which are considered the golden standard for the diagnosis of CAI [19, 20].

Subjects participated in one overnight video-polysomnographic study. Basic recordings of the polysomnography included standard EEG with six derivations, electrooculogram and electrocardiogram. Other measurements included electromyogram (chin and tibial muscles), nasal–oral flow, thoracic and abdominal respiratory efforts, oxyhemoglobin saturation, sound and body position. No transcutaneous PCO2 was measured. Sleep staging was scored using the American Academy of Sleep Medicine manual or Rechtschaffen & Kales for the scoring of sleep and associated events [21, 22]. All events were calculated per hour of sleep (index). In 11 of the PWS children, at least one PSG was performed before or shortly after ITT or GT (mean time between PSG and the stimulation test: - 0.37 ± 0.39 years). PSG data were retrieved from the respective archives and reviewed by the same pediatric neurologist (KD) specialized in sleep disorders, in order to calculate the CA and OA index. An OA index equal to or less than 1 per hour and a CA index of ≤ 0.9 per hour were considered as normal [23].

Results were statistically analyzed with the Statistical Package for Social Sciences (SPSS 20.0, Chicago, IL). Results are presented as median (95 % confidence interval (CI95%)) calculated according to Hodges-Lehmann or as mean ± SEM or as %. To test if the variance of the cortisol levels was different between the two groups (PWS and controls) and between the two stimulation tests (ITT and GT), the O’Brien test for the homogeneity of the variance was applied. Comparative analyses were done using the Mann Whitney test and correlation analysis using non parametric Kendall’s tau correlation. A *p*-value <0.05 was considered significant.

The study was approved by our local medical ethical committee of UZ Ghent university hospital (B.U.N.143201112296).

## Results

### Clinical characteristics

At GH testing, PWS patients were slightly but not significantly younger than the controls (respectively, median (CI 95 %) age: 5.6 (3.8;8.3) vs. 8.2 (6.6;10.2) years; *p* = 0.12). As expected, BMI-Z-score was higher in the PWS group than in the control group (BMI z-score respectively, median (CI 95 %): 1.65 (0.5; 2.7) vs. -0.65 (-1.11; -0.15) kg/m^2^, *p* = 0.0001). Nine (45 %) of the patients were boys in the PWS group and 16 (48 %) in the control group.

### GH status

In the PWS group (Table 1), six patients (30 %) had undergone an ITT, thirteen (65 %) a GT, in one child (# 14), both ITT and GT had been performed. ITT and/or GT was suggestive of GH deficiency in 50 % of the PWS children. The median (CI 95 %) age at start of GH therapy was 5 (3.7; 6.6) years.

**Table 1:** Summary of PWS patient characteristics and overview of their laboratory and PSG results

#	Age (y) at start of GH	GH Status	Age (y) at the test	ITT/GT	Peak cortisol (μg/dl)	Cortisol increase (μg/dl)	Δ (y) age at PSG and age at test	BMI z-score at PSG	CA index	OA index	Tonsil-lectomy
1	1,9	normal	1,7	GT	30,78	23,07	-	-	-	-	-
2	2	GHD	1,8	GT	41,98	32,17	−0,76	−0.5	0,7	0	1
3	3,8	normal	2,9	GT	26,5	9,0	-	-	-	-	-
4	5	normal	3,3	GT	20,6	12,6	-	-	-	-	-
5	3,4	GHD	3,4	GT	39,3	27,3	0,34	3.6	0,1	15	1
6	5,4	GHD	5,4	GT	31,4	18,7	1,71	2.9	0,1	13,4	1
7	12,1	normal	12	GT	20,2	15,84	-	-	-	-	-
8	8,5	GHD	14,4	ITT	16,6	9,97	−1,82	1.4	0,6	0,4	3
9	1	normal	0,8	GT	38,32	18,75	−0,22	−2.1	1,9	0,1	3
10	4,2	GHD	3,5	ITT	33,11	13,07	0,5	5.7	5,1	0	3
11	3,8	GHD	3,7	GT	47,1	19,9	0,05	−0.2	0,7	0,5	1
12	5	normal	4,4	ITT	23,45	16,4	-	-	-	-	-
13	5,2	normal	4,7	ITT	30,83	20,07	-	-	-	-	-
14	6,9	normal	5,5	GT	26.3	19.44	-	-	-	-	-
			5,6	ITT	24.0	16.92	-	-	-	-	-
15	2,9	normal	5,9	GT	31,5	24,69	−3,08	−0.8	0	4,2	3
16	6,2	GHD	6	GT	26,34	18,28	0	0.9	2,8	0,3	3
17	5,5	GHD	7,7	ITT	26,82	22,9	0,27	3.9	0,6	0,2	2
18	NA	GHD	9,4	GT	23,6	17,09	-	-	-	-	-
19	13,9	normal	12,2	ITT	29,3	12,5	-	-	-	-	-
20	5,4	GHD	14,7	GT	20,88	17,33	−1,02	3.1	1,2	0,5	3

*y* years, *GH* Growth Hormone, *ITT* insulin tolerance test, *GT* glucagon test; cortisol increase was calculated between the lowest cortisol and the highest cortisol level during the stimulation test, *PSG* polysomnography, *BMI* body mass index, *CA index* central apnea index (#/hour), *OA index* obstructive apnea index (#/hour), *NA* not applicable; tonsillectomy:1 = performed after PGS, 2 = before PGS, 3 = no tonsillectomy

### PSG results

Eleven of these children had undergone a PSG on average 0.37 ± 0.39 years before the GH stimulation test (Table 1). Four of them exhibited an increased CA index in comparison to the others of the group.

### Cortisol response

In one (# 8) out of twenty PWS patients, CAI was suspected, based on an insufficient cortisol peak value of 16.6 μg/dl after ITT. The only child with CAI had a CA index similar to the others of the PWS children, whereas the four PWS children with a raised CA index reached a sufficient cortisol response during the stimulation test (Table 1).

**Fig. 1:**
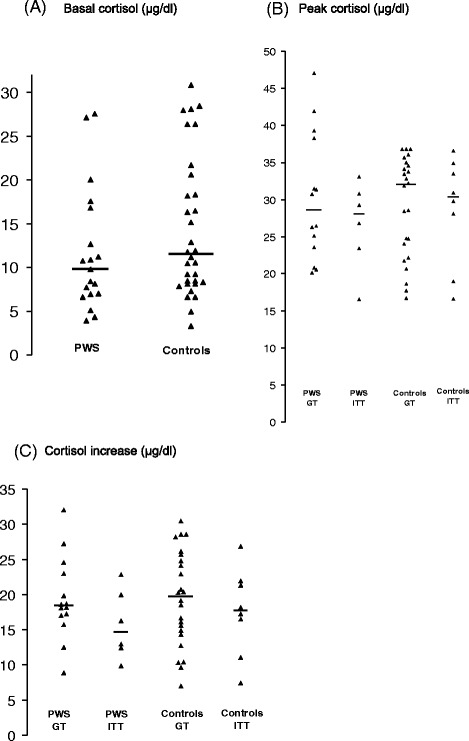
**a** basal, **b** peak cortisol levels and **c** cortisol increase in PWS and control children after a glucagon (GT) or an insulin tolerance test (ITT). The black lines represent the medians

**Fig. 2:**
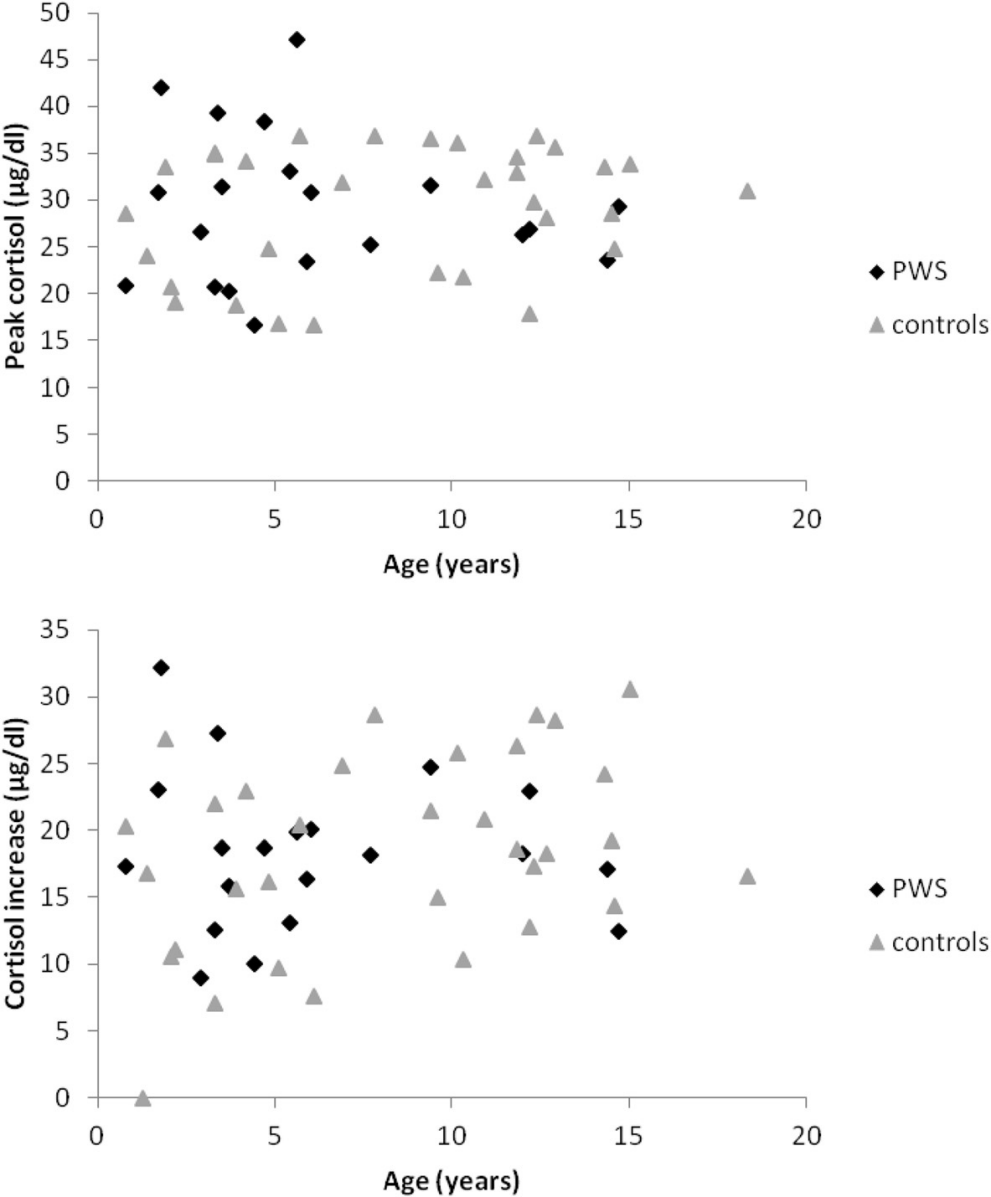
Correlation between peak cortisol levels (*upper panel*) and cortisol increase (*lower panel*) and age at the stimulation test in PWS (*black square*) and control (*grey* *triangle*) children

**Fig. 3:**
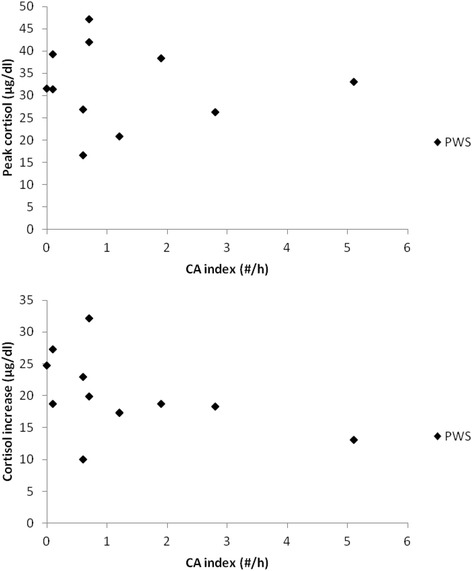
Correlation between peak cortisol levels (*upper panel*) and cortisol increase (*lower panel*) and central apnea (CA) index in PWS children

Basal cortisol levels did not differ between the PWS and the control children (*p* = 0.20) (Fig. 1a). Peak cortisol levels (Fig. 1b) and cortisol increase (Fig. 1c) were also not significantly different between the two groups (respectively *p* = 0.7 and *p* = 0.64). In both the PWS and control group, no difference was observed between the GT- or the ITT-induced peak cortisol response (respectively, *p* = 0.57 and *p* = 0.73) or cortisol increase (respectively, *p* = 0.24 and *p* = 0.63). Therefore results of both tests were combined for the correlation analysis. The variance of the cortisol levels was not statistically different between the two groups (PWS and controls) and between the two stimulation tests (ITT and GT) (PWS versus control group: basal cortisol levels, *p* = 0.55; peak cortisol levels, *p* = 0.505; cortisol increase, *p* = 0.57; ITT versus GT, even after adjustment for the group: basal cortisol levels, *p* = 0.44; peak cortisol levels, *p* = 0.49; cortisol increase, *p* = 0.16). No significant correlation between peak cortisol levels or cortisol increase was found with gender, BMI-z-score, GH peak or GH status neither in the PWS group nor in the control group (Table 2). However, the cortisol peak value showed a significant inverse correlation with age in both PWS and control children (Fig. 2) (respectively, *p* = 0.012 and *p* = 0.0067). The decrease with age of the peak cortisol levels after stimulation test was similar in the two groups (*p* = 0.13). A similar inverse correlation between cortisol increase during the stimulation test and age was present but only significant in the control group. A significant relationship between cortisol response and CA index (Fig. 3), or other PSG parameters in PWS children could not be demonstrated.

**Table 2:** Correlation of peak cortisol levels and cortisol increase in PWS and control children

*p*=	PWS		Controls	
	Peak cortisol	Cortisol increase	Peak cortisol	Cortisol increase
Age	0.012*	0.64	0.0067*	0.058*
Sex	0.99	0.99	0.38	0.25
BMI-z-score	0.83	0.83	0.10	0.90
GH peak	0.53	0.88	0.40	0.56
GH status	0.43	0.43	NA	NA
CA index	0.94	0.14	NA	NA
OA index	0.75	0.64	NA	NA

*GH* Growth hormone, *GH* status: normal or GH deficiency, *CA* central apnea, *OA* obstructive apnea, *NA* not applicable

*p < 0.05

## Discussion

Our data, based on peak cortisol and cortisol increase after ITT or GT, showed an insufficient cortisol response in only one out of twenty PWS children (5 %). We could not replicate the hormonal findings by de Lind van Wijngaarden et al. [10] who found CAI to be present in 60 % of PWS children, based on a metyrapone test. Our results are in line with the results from Nyunt et al., Corrias et al., Grugni et al. and Farholt et al. [12–15], who found CAI in respectively 0, 4.8, 7.5 and 0 % of the PWS patients. The optimal test for evaluating central adrenal insufficiency in children is debated and the discrepancies in the prevalence of CAI between studies may be due to the different kind of tests used. The low prevalence of CAI reported by Nyunt et al., Corrias et al. and Grugni et al. may be due to the lack of sensitivity of the LDST they used to diagnose CAI: only 50 % of the patients with an ACTH deficiency, based on metyrapone tests showed an insufficient cortisol response when tested with LDST [24, 25]. On the other hand, as compared to ITT, the metyrapone test with an ACTH cut-off of 33 pmol/l as it was used by de Lind van Wijngaarden et al. [10] yielded a high false-positive rate (specificity 47 %) [26]. In fact, ITT remains the gold standard test for evaluating central adrenal insufficiency in children [19, 20]. Given its possible complications, the GT is considered an equal and safe alternative and yielding similar cortisol responses [18, 27, 28]. In our study, based on these tests, we could not confirm the high prevalence of CAI in PWS children.

We did not find any significant correlations between cortisol response and PSG parameters and, in particular, the CA index. Thus, our results do not support the hypothesis of a link between CAI and SRBD, as suggested by de Lind van Wijngaarden et al. [11]*.* Moreover, in our study, the only child with CAI was 14.4 years old when tested. As shown by others in PWS patients younger than 17 years of age [13, 14], the peak cortisol after stimulation decreased in function of age. In our study, this inverse correlation was also found in controls, and thus is probably not related to the pathophysiology underlying PWS. Sudden unexplained deaths in PWS have been described to occur more frequently at a young age [9]. We did not observe abnormal stress-induced cortisol responses in young PWS patients. Therefore, a causal link between sudden death and CAI as suggested by de Lind van Wijngaarden et al. [10] seems to be unlikely from our study.

Our study has methodological limitations due to its retrospective design and multicenter data collection. Although a large variability in cortisol levels was found, the dispersion of the cortisol levels was not different in PWS as compared to controls or in the GT as compared to the ITT. The lack of a standard cortisol assay method may explain some of the variability in the cortisol levels reported in this study. Indeed, Kaslaukaité et al. have shown in a meta-analysis that, due to the lack of cortisol assay standardization, the error in measuring cortisol can be up to 6 μg/dl (165 nmol/l) between studies [19]. Moreover, a variability in peak cortisol response to insulin-induced hypoglycemia is a common finding and an average variability of 8 to 12% has been reported by Vestergraard et al. [29]. A suboptimal cortisol response to a glucagon stimulation test [30] has also been reported in 8 % of healthy individuals. The sample size was small, both due to the rarity of the condition and to the lack of a standardized protocol for the diagnosis and management of PWS patients with regard to diagnosis of CAI and/or SRBD in Belgium. However, by combining the data obtained after ITT and GT, and by including a large control group, our study reached sufficient power to reliably estimate the prevalence of CAI in our patients and to investigate correlations between cortisol response and PSG parameters.

## Conclusion

CAI is a rare phenomenon in PWS. Our data do not support the theory of an overarching hypothalamic dysfunction resulting in both hypothalamic-pituitary-adrenal axis and central respiratory dysregulation. A causal link of CAI with the reported sudden, unexplained deaths at a young age is unlikely.

## Abbreviations

PWS: Prader-Willi Syndrome
CAI: Central adrenal insufficiency
ITT: Insulin tolerance test
GT: Glucagon test
GHD: Growth Hormone deficiency
PSG: Polysomnography
GH: Growth hormone
CA: Central apneas
OA: Obstructive apneas
SRBD: Sleep-related breathing disorders
LDST: Low-dose ACTH test
HDST: High-dose ACTH test
